# The dynamic interdependencies among the negativity and the positivity in news and user-generated content about safety in a firm’s products and the firm’s product recalls

**DOI:** 10.1371/journal.pone.0305287

**Published:** 2024-08-08

**Authors:** Vivek Astvansh, Yen-Yao Wang

**Affiliations:** 1 Associate Professor of Quantitative Marketing and Analytics, Desautels Faculty of Management, McGill University, Montreal, Canada; 2 Bensadoun School of Retail Management, McGill University, Montreal, Canada; 3 Adjunct Associate Professor of Data Science, Luddy School of Informatics, Computing and Engineering, Indiana University Bloomington, Bloomington, IN, United States of America; 4 Affiliate, Environmental Resilience Institute, Indiana University, Montréal, QC, Canada; 5 Department of Business Analytics and Information Systems, Harbert College of Business, Auburn University, Auburn, AL, United States of America; University of Almeria: Universidad de Almeria, SPAIN

## Abstract

This article examines the dynamic interdependencies among the negativity and the positivity in news and user-generated content about safety in a firm’s products (or the lack thereof) and the firm’s product recalls. The authors use a panel vector autoregression (PVAR) to unearth theoretically novel and managerially relevant asymmetric associations. Specifically, they find that the negativity in the news negatively correlates with recalls, whereas the negativity in UGC positively correlates with recalls. Whereas the positivity in the news positively correlates with recalls, the positivity in UGC does not matter. Further, the negativity in the news and the negativity in UGC substitute for each other, whereas their positive counterparts complement each other’s associations with recalls. Lastly, the negativity and positivity in the news have significant, though differently patterned, long-term associations with recalls. The findings contribute to research on the associations between earned media and managerial decisions in the product market.

## Introduction

A product recall is “an organization’s actions—legally mandated or voluntary—intended to prevent from use consumer goods that have a safety defect and/or are noncompliant with regulations” [1]. Product recalls are consequential [2–10]. While extant research on recalls has provided managerial insights, little effort has been sought to study the dynamic relationships among news organizations, the public—two stakeholders for whom safety failures in a firm’s products are highly relevant—and the managerial decision of the number of recalls. These two stakeholders are intertwined as they create and distribute *earned media* (news and user-generated content [UGC], respectively). Interestingly, news organizations and the public can frame their content negatively, emphasizing the problem (defects) [11–13], and/or positively, highlighting the solution (recalls) [14]. Indeed, anecdotes support this coexistence of negativity and positivity in news and UGC about the safety of a firm’s products [15, 16]. We thus consider the negativity and the positivity in news and UGC to empirically examine the interdependencies among the negativity/positivity in news and UGC about safety in a firm’s products and the firm’s number of recalls.

Our theoretical premise is as follows. We reason that managers may interpret the negativity in earned media (news and UGC alike) as reputational costs [17–20], nudging them to take actions that lower these costs. That is, the negativity in earned media may negatively correlate with the number of units the firm recalls in the following period. Interestingly, the opposite hypothesis is equally plausible. Safety regulators [21, 22] and product liability lawyers [23, 24] are known to use negative news and UGC to determine how responsive and responsible a firm has been in addressing safety in its products. To the extent that negative content is triggered by incidents of product-caused consumer harm [25, 26], managers may use the content to discover defects [27–30] and recall more units in the following period, appearing responsive and responsible. Just as managers may interpret negativity in earned media as reputational costs, they may interpret positivity in earned media—that too in the face of product safety—as reputational benefits. Thus, we reason that these reputational benefits positively correlate with the number of units recalled in the following period [31].

We tested our premise in the context of automobile recalls initiated by 22 manufacturers in the United States. We looked at each month between June 2009 and 2015 for 1,483 manufacturer-month observations. For each observation, we obtained the number of recalled vehicles (our measure of “recalls”) from the National Highway Traffic Safety Administration (NHTSA), the text of all relevant news articles (relevant means that the articles included keywords such as “defect” and “faulty”) from Factiva (totaling 19,812 articles), and the text of all relevant tweets (tweets serve as our proxy for UGC) (totaling 164,944 tweets). We used the Linguistic Inquiry and Word Count (LIWC) computer program [32, 33] to measure the levels of negativity and positivity in each news article and each tweet.

To examine the dynamic interdependencies in the news, UGC, and product recalls, we leverage panel vector autoregression (PVAR). Prior research in finance (e.g., [34]), marketing (e.g., [35–39]), and information systems (e.g., [40–42]) has used a PVAR to examine dynamic interdependencies in various settings. Following this stream of research, we use a PVAR to examine the short- and long-term dynamic relationships among our five main variables: the negativity in the news, the positivity in the news, the negativity in UGC, the positivity in UGC, and the number of units recalled in a month.

In modeling the dynamic associations of earned media with a firm’s recalls—while accounting for reverse causality and endogeneity of the main variables in our system of equations—we offer robust evidence to managers. Specifically, after controlling for several variables that have been shown to predict a firm’s recalls (e.g., [43, 44]), we document that the negativity in news about safety in a firm’s product in a month negatively correlates with recalls in the following month. In contrast, the positivity in the news positively correlates with recalls. In contrast, the negativity in UGC about a firm’s product defects in a month positively correlates with recalls in the following month. Contrary to our expectations, the positivity in UGC does not have a statistically significant association.

Recent management research has offered arguments for the interplay between news and UGC, drawing distinctions between the broader set of a firm’s *stakeholders* and the *public*. Stakeholders refer to institutional investors, suppliers, business partners, and employees who regularly transact with the firm, thus more directly impacting its success or the lack of it. In contrast, the public refers to members of the civil society ([45]), comprising “thousands of current and potential customers, employees, or individual investors, who may be encouraged or discouraged to buy from, work for, and invest” in the firm ([46]: 223). For example, Blevins and Rogazzino [47: 220] contended that news is “more relevant for understanding [key] stakeholders’ view of organizational reputation,” whereas social media content is “more relevant for understanding the public domain’s view of organizational reputation.” Relatedly, Etter et al. [48: 29] argued that “social media now give voice to actors who previously had limited access to the public domain, and they enable these actors to bypass the gatekeeping function of traditional news media and reach wide audiences connected through online social networks.” According to Blevins and Ragozzino [47: 221], “Traditional media’s role in influencing organizational reputation is now even stronger… this is a direct consequence of social media’s growth.” We leverage these arguments to explore whether news and UGC complement or substitute for each other in influencing recalls. “Substitute” means that UGC (news) would decrease the association between news (UGC) and recalls. “Complement” means that UGC (news) would increase the association between news (UGC) and recalls. The results suggest that the negativity in the news and that in UGC substitute for, whereas their positivity complements each other in impacting recalls. In addition, using impulse response functions (IRFs), we document the asymmetry in direction and temporal persistence of the negativity in news and the positivity in the news on recalls. These findings can help managers design temporally segregated strategies to moderate the associations. Lastly, following recall research [11, 44, 49], we examine whether the associations of news and UGC on recalls are contingent on the managerial discretion in making the recall decision, as manifest in the low vs. high severity of the underlying defect. Although not the focus of our research, our PVAR also helps managers understand (1) how recalls may correlate with subsequent news and UGC about the firm’s product defects and (2) the relationship between two types of earned news in the recall setting. Such consideration of the dynamics and interdependencies among the five main variables—the negativity in the news, the positivity in the news, the negativity in UGC, the positivity in UGC, and the number of units recalled—helps illustrate the complexity of the interaction between the two types of earned media and subsequent managerial decisions in the product market.

Our primary contribution is to the literature at the intersection of media and marketing decisions. Although this literature distinguishes between earned media (i.e., news) and paid media (i.e., advertising), it has paid relatively less attention to UGC as another form of earned media ([37, 40, 50, 51] are notable exceptions). By studying the *dynamic* relationships between news and UGC as two types of earned media and managers’ endogenous decisions of recalls, we unearth theoretically novel asymmetries between the two types and their negative/positive valences. In addition, we document the complementary associations of the negativity in news and the negativity in UGC, and the substitution associations of their positivity counterparts. Last, in showing the relationship between UGC and managers’ endogenous choice of recalls in a period, our article contributes to the emerging evidence on the association of UGC with aggregate organizational outcomes [52–57].

Section 2 overviews the relevant research on product recalls, and the role of news and UGC in conferring reputation to a firm. Section 3 describes the theoretical arguments in support of our hypotheses. Section 4 narrates our data collection and discusses the PVAR methodology. Section 5 presents the results. Section 6 concludes with a discussion of our findings’ theoretical and managerial implications and the limitations of our research that merit future attention.

## Relevant literature

### Product recall

Product recall represents safety failures in a firm’s products [58]. Academics in strategic management, marketing, and operations management have extensively examined the causes of a firm’s product recalls, mostly by analyzing secondary data. The dependent variable (DV) is usually a firm’s number of recalls or the number of units recalled in a period [43, 44, 59–61]. The causes (independent variables) have been discussed from the focal firm’s and/or its product-market stakeholders’ perspective [2], such as the deployment of marketing assets [62] and marketing personnel [63], CEO characteristics [64], the levels of the recalled product [43, 49, 61], manufacturing operations [59, 61], supply chain [65, 66], the board of directors [44], the industry [67], and the regulatory investigation [4]. Notable in this list are two omissions. First, recall researchers have overlooked the relationship between news organizations and a firm’s recalls ([11] is a welcome exception). Second, recall researchers have not examined the role of UGC on a firm’s recalls. Our research rectifies these omissions.

### News and media reputation

News media refer to professional news *organizations* that produce and deliver news to the public. Academics in communications, journalism, and sociology disciplines have long studied news organizations’ role in society (e.g., [68, 69]), while business academics have focused on news organizations’ role *in business* (e.g., [20, 70]).

Management academics have theorized that news about a firm constitutes the firm’s *media reputation*, that is, “the overall evaluation of a firm presented in the [news] media” [70: 1091]. “Providing institutional and cultural accounts within which the appropriateness and desirability of [a firm’s] actions can be evaluated… affects impression formation and legitimation of firms” [71: 632]. News organizations serving as conduits of institutional prompt “firms to conform to prevailing institutional logics” [17: 137].

Importantly, news organizations leverage not only the volume of coverage but also the evaluative tone of this coverage (i.e., negative/positive valence in the presentation of the information) to shape stakeholders’ perceptions of firm actions and inactions [72–74]. When the focal issue is of public interest (e.g., a safety defect in a product), news organizations’ evaluative tone becomes particularly consequential [75, 76]. Further, given the fact that the media’s need to tell a “story” [77] and the tendency to sensationalize [78] the stories they tell often portray a firm’s managers as characters, elevating them to celebrity status [79], particularly in the event of positive news. Conversely, since negative news spills over—perhaps more easily and more often than its positive counterpart—to a firm’s managers, when negative news occurs, it will raise doubts about their credibility and integrity [80].

By deciding how to disseminate news of managers’ actions and inactions, the media dictate how much a firm’s owners and participants in the managerial labor market learn about the managers [81, 82]. Further, by characterizing the managers’ actions as positive and/or negative, the media shapes the perceptions of the actions and inactions [81, 82]. Empirical evidence supports these arguments. For example, positive news about a firm often decreases the independence of the firm’s board [17] and the adoption of board reform practices [20], whereas the negativity in news hurts a manager’s compensation [83, 84] and future employment prospects [82] and increases the likelihood of forced turnover [83]. Managers are aware of this potential influence and thus make decisions that serve the best interests of the firm’s owners and other stakeholders [82, 85]. The number of units recalled in a month—our DV—is one such decision.

The news of safety in a firm’s products may follow either or both of the following two trajectories. First, news organizations may emphasize the safety defect and its consequences for the public (negative coverage) [75]. Second, and perhaps surprisingly, these organizations also “may portray the firm in a positive light” [14: 768], positioning recall as a brand’s corrective [86] and socially responsible [14] response to the unsafe product (positive coverage). Indeed, although intuition suggests that media coverage would be predominantly negative in the context of product defects, academics have discovered that coverage of such problems may have a positive valence (e.g., [13]). As Shipilov et al. [20] reasoned, “when analyzing media coverage, positive coverage is different from the absence of media coverage” (p. 1372). Stated differently, the valence of news—whether negative or positive—has distinct properties that make the levels of negativity and positivity separate dimensions [87–89]. Following management research, we consider both dimensions and offer theoretical assertions to examine the relations among the negativity in the news, the positivity in the news, and recalls.

### User-generated content and public reputation

Social media refers to “new information and communication technologies that enable users to connect and publicly exchange experiences, opinions, and views on the Internet” [90: 29]. The content on social media platforms may be generated by either the individual users of the platform, creating UGC, or by the firms that create an account/page on a platform, creating firm-generated content (FGC) [42].

Management academics theorize that just as news establishes a firm’s media reputation, UGC reflects its *public reputation*—“the public’s perception or impression of an organization, usually associated with a given action or event” [91: 66]. For a firm, then, UGC holds a diagnostic function, with its public reputation becoming more consequential when it faces an adverse situation (e.g., [55, 57]). Indeed, UGC is “changing how evaluations of the quality, competence, or character of organizations are produced, disseminated, and accessed in the public domain” [48: 28]. In highlighting the role of UGC in quality control and managerial decisions, Dellarocas [92: 1409] noted that UGC can “accelerate the dissemination of information about product defects” and “can also act as a powerful disciplining mechanism.” Such theoretical arguments enable us to hypothesize the dynamic relationships between news and UGC in a context relevant to news organizations and social media users. Although researchers have paid attention to the role of UGC in discovering product defects [27, 28], responding to a corporate social irresponsibility event [93], and aggravating stock returns in response to a supply-chain glitch [94], an empirical assessment of the associations of UGC on a firm’s operational decisions (e.g., the number of units recalled in a period) remains unexplored.

In line with the management academics’ consideration of the negativity and the positivity of news as two variables, we separately consider the level of the negativity and the level of the positivity of UGC and propose how and why they may correlate with recalls. We next offer our theoretical assertions about the interdependencies between the negativity and the positivity in the news and product recalls, as well as parallel hypotheses about the associations of UGC.

## Hypotheses

### The association of negativity in news and UGC about safety in a firm’s products with the firm’s product recalls

We reason that the negativity in the news and UGC about the safety of a firm’s products makes managers perceive three types of reputational costs: economic, social, and psychological [17–20]. First, the negativity signals managers’ inability to shape public perceptions of their firm, damaging their reputations in the eyes of future employers [18, 81, 95–98]. Second, the negativity may also damage the managers’ reputations within their communities; in extreme cases, leading to embarrassment, shame [99], and stigmatization [100, 101]. Third, criticism from news organizations and social media users can reduce managers’ self-confidence and evoke negative emotions (such as anger, annoyance, and fear) [74, 102–104]. These reputational costs nudge managers to lower the incidence of decisions that might further damage their reputations in the eyes of news organizations, and, by extension, the firm’s stakeholders (i.e., business customers, suppliers, partners, investors, and employees) [17].

*H*_*1*_: *The* ***negativity*** *in the* ***news*** *about safety in a firm’s products negatively correlates with its product recalls in the following period*.
*H*_*2*_: *The* ***negativity*** *in* ***UGC*** *about safety in a firm’s products negatively correlates with its product recalls in the following period*.

Interestingly, at least two reasons make the competing hypotheses equally plausible. First, to the extent that negative news and UGC are triggered by product-harm incidents, they may help the firm diagnose the defect and determine a solution. Indeed, firms are known to mine news and UGC [25, 26] to detect safety defects in products [27–30]. Further, because regulators [21, 22] and product liability lawyers [23, 24] are also known to use UGC to determine how proactive/reactive a firm has been in initiating recalls, managers are likely to mitigate the possibility of regulatory and legal costs by being responsive and recalling more units in the following period [45, 91].

Second, research on the disciplining power of media [18, 83, 105, 106] documents that media and public criticism can remind managers of their social responsibility. Specifically, managers’ vulnerability to media criticism can make them view recalls as socially responsible decisions, and thus, they may respond to the negativity with an increase in recalls [107]. Such managerial response can also be self-serving if it enables the managers to cultivate goodwill in the eyes of the media. Such goodwill may come in handy if the firm were to run against a safety regulator or contest a product liability lawsuit [108]. Empirical evidence supports this theoretical argument. For example, the negativity in news and public opinion about a firm increases its philanthropy spending [107] and its adoption of a CSR reporting standard [109] and stops it from polluting the environment [110]. At an aggregate level, the negativity in media makes a country’s private sector more responsive to environmental issues [95]. We thus have reason to expect that managers may respond to the negativity in the news and UGC by increasing recalls.

*H*_*1alt*_: *The* ***negativity*** *in the* ***news*** *about safety in a firm’s products positively correlates with its product recalls in the following period*.
*H*_*2alt*_: *The* ***negativity*** *in* ***UGC*** *about safety in a firm’s products positively correlates with its product recalls in the following period*.

### The association of positivity in news and UGC about safety in a firm’s products with the firm’s product recalls

While commenting on a *Time* magazine report on why recalls have become more common, an expert opined, “Most recalls could even be a good thing” [16: 1]. Similarly, a *Consumer Reports*’ assessment of “the truth about car recalls” found that contemporary “cars are actually safer” [15: 1]. As these anecdotes illustrate, even in the face of safety in a firm’s products, earned media “may portray the [recalling] firm in a positive light” [14: 768] by framing the recall in terms of a firm’s diligence about quality issues [111], and as a corrective [86] and a socially responsible [14] response to the unsafe product. Indeed, although one might assume that media would be predominantly negative in the context of product defects, researchers have gained unexpected insights by considering the positivity of earned media [13]. When news organizations and the public discuss a firm positively despite the negativity associated with product safety, the positivity deflects attention away from a firm’s wrongdoing (e.g., safety defects). Instead, it emphasizes the firm’s positive response (e.g., recall) [13]. Such positivity reflects the media’s belief that a firm is acting appropriately by accepting responsibility for its unsafe products and effectively executing corrective actions [70, 71, 112].

We earlier reasoned that criticism by news organizations and the public makes managers perceive reputational costs. Conversely, praise by these two stakeholders in the face of a predominantly negative context of safety defects makes managers accrue reputational benefits [120]. Such perceived reputational benefits may infect managers with hubris [113, 120], which may nudge managers to make riskier decisions [120]. Recalls are known to damage a firm’s performance in the product market (e.g., sales, [151] and financial market (e.g., stock return, [43]), and are thus riskier decisions. Therefore, managerial hubris may lead them to recalling more units in the next period. In addition, managers are likely to view praise as an external confirmation of their product’s reputation for high quality. The reputation-as-asset logic [20] suggests that managers may conclude that a positive reputation for quality can help the firm withstand criticism [13, 79]. They may thus consider appropriating the reputation by slipping in a few additional units in recalls in the following period [113].

*H*_*3*_: *The* ***positivity*** *in the* ***news*** *about the safety of a firm’s products positively correlates with its product recalls in the following period*.
*H*_*4*_: *The* ***positivity*** *in* ***UGC*** *about safety in a firm’s products positively correlates with its product recalls in the following period*.

### The interactional association of news and UGC about safety in a firm’s products with the firm’s product recalls

Between 25% and 77% of people in different countries use social media as a source of news [114, 115]. In the United States, social media platforms have outpaced print newspapers as a news source [116]. These statistics suggest a strong and dynamic relationship between news and UGC, though research on this relationship is limited (see [50, 117] for two notable exceptions). Further, commentators and researchers are divided on whether news and UGC complement or substitute for each other. Sismeiro and Mahmood [117] showed that in the aftermath of a four-hour outage on Facebook, news websites received fewer visitors, suggesting that Facebook brings additional traffic to news websites (complementary association) rather than reduces such traffic (substitution association). In a more recent study, Jiao et al. [50] found that the volume of news on a firm decreased its idiosyncratic stock risk and trading volume, whereas the volume of UGC on a firm *increased* such risk and trading volume.

Jiao et al. [50] have contended that news represents genuine information. Thus, a high volume of news on a firm dampens its investors’ disagreement about its stock’s value, decreasing idiosyncratic risk and trading volume. In contrast, the researchers have posited that the high volume of UGC about a firm suggests disagreement among the public, increasing idiosyncratic risk and trading volume. They have also demonstrated that news causes UGC, whereas UGC does not cause news, a finding that runs counter to prior evidence [51, 146]. The finding also runs counter to anecdotal evidence of news organizations curating stories from social media platforms, in which politicians’ and celebrities’ tweets and Instagram posts are presented as news [118, 119]. Our analysis builds on this dynamic interaction between news and UGC.

In reasoning that the negativity in news and UGC poses economic, social, and psychological costs to a firm’s managers, we asserted that the negativity in news and UGC may negatively correlate with the number of recalled units in the following period to help managers lower these costs. On the other hand, to the extent that the negativity is triggered by product-harm incidents, it may help the firm diagnose defects and lower potential regulatory and legal costs.

Now, consider the likely scenario in which negative news and negative UGC co-exist. We reason that the negativity in UGC would make the three costs of the negativity in the news salient to managers, strengthening the latter’s negative association with recalls. Reciprocally, negative news would increase the salience of the firm’s key stakeholders to managers, thus lowering managerial attention to concerns about consumer safety, as reflected in UGC. That is, negative news would weaken the positive association of negative UGC with recalls. We thus hypothesize that the negativity in the news and the negativity in UGC substitute for each other in associating with a firm’s recalls.

*H*_*5*_: *The* ***negativity*** *in the* ***news*** *and the* ***negativity*** *in* ***UGC*** *about safety in a firm’s products have a substitution association (negative interaction) on its product recalls in the following period*.

Next, we consider the interaction association of the positivity in news and that in UGC. We earlier contended that the positivity in news buoys managerial hubris, nudging managers to make riskier decisions [113, 120], such as recalling more units in the following period. On the other hand, the positivity in UGC about product defects is a testament to the firm’s reputation for high-quality products. Such testimony also leads to managerial hubris. When present in unison, the positivity in the news and the positivity in UGC instill a sense of exaggerated hubris in a firm’s managers. Such collective praise from both types of earned media would strengthen managerial hubris. Accordingly, we hypothesize a complementary association between the positivity in news and the positivity in UGC on a firm’s recalls.

*H*_*6*_: *The* ***positivity*** *in the* ***news*** *and the* ***positivity*** *in* ***UGC*** *about safety in a firm’s products have a complementary (positive interaction) association on its product recalls in the following period*.

## Data and methodology

### Research context

We chose the U.S. automobile industry as the context for the following reasons. First, considering the widespread occurrence of recalls in the automobile industry, our research gains greater relevance with the industry [121]. Second, given the economic importance and public interest in the U.S. automobile industry [122], there has been a notable increase in news coverage and UGC increase within this domain [12, 123], rendering it pertinent for assessing the efficiency of news and UGC (e.g., [42]). Thirdly, by concentrating on a single industry, the necessity for encompassing a broad spectrum of cross-industry factors to mitigate potential heterogeneity in multi-industry studies diminishes, thereby enhancing the internal validity of findings [124].

We concentrate on two forms of earned media: news and UGC, particularly on safety-related discussions concerning the focal firm’s products. Management scholars have theorized and provided empirical evidence regarding the effects of negative [100] and positive news on firm outcomes [125–128]. Twitter is a prevalent platform for UGC, offering users a rapid means to encounter fresh and trending content. Previous research has highlighted the efficacy of UGC sourced from Twitter, particularly in response to adverse situations (e.g., [94]), owing to its unique characteristics. Furthermore, due to the spontaneous, passionate, information-rich nature of UGC, prior research has also demonstrated the influence of UGC on firm outcomes in the context of product recall (e.g., [35, 129]). Nevertheless, our comprehension of the relationship among news, UGC, and a firm’s recalls remains considerably limited. Our research endeavors to address this gap in understanding.

### Data

The data collection process comprised six steps. Initially, we referred to Ward’s Intelligence database to acquire the roster of 22 passenger car manufacturers representing 95% of the yearly sales volume of passenger cars in the United States. These manufacturers are Acura, Audi, BMW, Buick, Cadillac, Chevrolet, Chrysler, Dodge, FIAT, Ford, Honda, Hyundai, Infiniti, Jeep, KIA, Lexus, Mazda, Nissan, Porsche, Subaru, Toyota, and Volkswagen. Next, we gathered car recall data from June 2009 to June 2015 (a six-year period) from the NHTSA’s recalls data file for each manufacturer. NHTSA is a federal agency within the U.S. Department of Transportation dedicated to enhancing transportation safety nationwide. It develops and enforces vehicle safety standards, investigates vehicle safety defects, and researches driver behavior and traffic. The NHTSA recall data is a part of its effort to improve transportation safety. This dataset is one of the primary sources for vehicle recalls and has been leveraged and validated in prior research (e.g., [3, 6, 35]). Our exercise on recall data collection yielded 207,489,321 recalled units. Third, we used a Python program to collect the text of user-generated tweets each month about safety in each manufacturer’s vehicles. Following prior research (e.g., [11, 42]), we used the combination of the manufacturer name and defect keywords—such as safety, recall, defect, and faulty—to obtain monthly user-generated tweets. This step yielded 164,944 tweets. In the fourth step, we applied identical search criteria on Factiva to compile the text of each distinct news articles on safety concerns in the products of each manufacturer. Factiva is a business information and research tool owned by Dow Jones & Company. It allows users to search, monitor, and analyze news and information to support research and decision-making processes. The reliability and validity of Factiva have been investigated extensively in prior studies (e.g., [3, 130, 131]). This step produced 19,812 news articles.

Fifth, we used the Linguistic Inquiry Word Count (LIWC) program [132] to measure the negativity and positivity in the news and UGC. LIWC was designed to evaluate text’s psychological and structural components. The tool has been widely adopted in psychology and linguistics [133]. LIWC calculates the proportion of words that match predefined dictionaries using word counts for a given text. LIWC includes a psychometrically validated internal dictionary comprising approximately 6,549 labeled words and word stems, each classified into one or more categories [132]. Several management researchers have used LIWC to measure news sentiment [13, 79, 134]. The reliability and validity of LIWC variables have also been investigated extensively in settings such as news [e.g., 37], online review (e.g., [135]), and UGC (e.g., [136]).

We briefly discuss how LIWC processes the text in the following. Upon receipt of a text sample, the software processes each word in the sample, one at a time. While processing each word, LIWC scans its dictionary file to find a match, and if a match is found, the corresponding category scale for that word is incremented. Following this process, a final score is assigned to each category, indicating the percentage of words in the text sample that align with that specific category.

Following prior management research, we used the LIWC variables “negemo” and “posemo” to measure the negativity and the positivity, respectively, in news articles and tweets. These two variables provide the percentage of positive (e.g., happy) and negative (e.g., angry, fear) emotion words in a text. Next, for each manufacturer-month, we computed a mean score for our four main variables—*the negativity in the news (Neg news)*, *the positivity in the news (Pos news)*, *the negativity in UGC (Neg UGC)*, and *the positivity in UGC (Pos UGC)*. Sixth, we collected a set of control variables from various validated sources, including complaints from the NHTSA complaints data file, public interest from Google Trends (see [137–139] for reliability and validity of Google Trends), sales and price information from the Ward’s Intelligence database, advertising expenditure from Kantar Media’s Ad$pender, the number of crashes involved each manufacturer’s vehicles from the NHTSA complaints data file, and the reliability score of each manufacturer’s models from *Consumer Reports*. Our final sample contains 1,483 manufacturer-month observations. The collection and analysis method complied with the terms and conditions for the source of the data. Table 1 presents variables’ definition, data sources, and summary statistics. Table A1 in the online S1 Appendix reports the Pearson pairwise correlation coefficients.

**Table 1:** Variable definitions and summary statistics.

Variable	Definition	#Obs.	Source	Mean	SD	Min	Max
Recalls	Number of vehicles recalled by the focal manufacturer in the focal month	1483	NHTSA	139911.9	543330.9	0	6700000
Neg news	Mean of the percentage of negative emotion words in news articles in the focal month about safety in and recalls of the focal manufacturer’s vehicles	1483	Factiva	1.29	1	0	4.82
Pos news	Mean of the percentage of negative emotion words in news articles in the focal month about safety in and recalls of the focal manufacturer’s vehicles	1483	Factiva	1.03	0.84	0	4.93
Neg UGC	Mean of the percentage of negative emotion words in user-generated tweets in the focal month about safety in and recalls of the focal manufacturer’s vehicles	1483	Twitter	2.79	2.3	0	25
Pos UGC	Mean of the percentage of positive emotion words in user-generated tweets in the focal month about safety in and recalls of the focal manufacturer’s vehicles	1483	Twitter	1.59	1.66	0	15
Complaints	The number of consumer complaints received in the focal month by the NHTSA about safety incidents involving the focal manufacturer’s vehicles	1483	NHTSA	250.06	347.91	0	5975
Public interest	An index of Internet search about the focal manufacturer in the focal month	1483	Google Trends	68.35	12.96	26	100
Volume of news	Number of news articles in the focal month about safety in and recalls of the focal manufacturer’s vehicles	1483	Factiva	13.36	42.54	0	875
Volume of UGC	Number of user-generated tweets in the focal month about safety in and recalls of the focal manufacturer’s vehicles	1483	Twitter	111.22	326.03	0	4360
Sales volume	The number of vehicles that the focal manufacturer sold in the focal month	1483	Ward’s	50357.22	53099.17	500	245239
			Intelligence				
Price	The average price of the focal manufacturer’s vehicles	1483	Ward’s	32749.86	13431.73	14192.5	90775
			Intelligence				
Ad spending	Dollars spent in the focal month by the focal manufacturer on advertising	1483	Kantar Media Ad$pender	4.20e+07	3.61e+07	238700	2.00e+08
Crashes	The number of crashes reported in the focal month and involving vehicles of the focal manufacturer	1483	NHTSA	11.25	27.32	0	689
Reliability	Mean reliability score of the focal manufacturer’s models in the focal year	1483	Consumer Reports	8.31	0.42	7.02	9.34

### PVAR specification

A vector auto-regression (VAR) model can examine models that entail undefined or challenging-to-define constraints, such as causality [41]. Leveraging the benefits of the VAR model and the structure of the panel data set, the PVAR offers several benefits to examine the dynamic interdependencies compared to traditional statistical analyses. It has been applied in prior research in multichannel communication (e.g., [36–38]) to examine interdependencies between multiple channels and firm decisions. We briefly discuss the benefits of PVAR, which are as follows: First, it treats the main variables as endogenous, making it well-suited for capturing dynamic interactions among variables without imposing ad hoc model restrictions, such as the exogeneity requirements imposed by other econometric methods [140]. Second, it models the dynamic interdependencies over time. Unlike traditional statistical analyses that may examine pairwise relationships, PVAR enables the detection of bidirectional relationships (i.e., feedback loops) among endogenous variables while ensuring the robustness of estimates against challenges such as non-stationarity, spurious causality, endogeneity, serial correlation, and reverse causality [141]. These unique characteristics enable a more nuanced understanding of how changes in one variable affect others in the system and vice versa, leading to a richer interpretation of the underlying dynamics. Third, it enables estimating variables’ long-term or cumulative effects through impulse response functions [35]. Fourth, the panel structure of our data provides the ability to manage unobserved individual (i.e., manufacturer) heterogeneity and employ instruments within the model, such as lagged DVs in the GMM (generalized method of moments) to tackle issues related to reverse causality and endogeneity, thereby attaining consistent estimates [34, 142].

Our PVAR specification is the following:

$$(\begin{array}{c} \;Recalls_{i,t}\\ \;Neg\;news_{i,t}\\ \;Pos\;news_{i,t}\\ \;Neg\;UGC_{i,t}\\ \;Pos\;UGC_{i,t} \end{array})=\sum_{s=1}^{p}\Phi_{s}(\begin{array}{c} \;Recalls_{i,t-s}\\ \;Neg\;news_{i,t-s}\\ Pos\;news_{i,t-s}\\ Neg\;UGC_{i,t-s}\\ Pos\;UGC_{i,t-s} \end{array})+\beta (\begin{array}{c} \;Complaints_{i,t-1}\\ \;Public\;interest_{i,t-1}\\ \;Volume\;news_{i,t-1}\\ \;Volume\;UGC_{i,t-1}\\ \;Sales\;volume_{i,t-1}\\ \;Price_{i,t-1}\\ \;Ad\;spending_{i,t-1}\\ \;Crashes_{i,t-1}\\ \;Reliability_{i,t-1} \end{array})+f_{i}+\xi_{t}+\varepsilon_{it}$$ (1)

*y*_*i*,*t*_ = (${Recalls}_{i,t},\;{Neg\;news}_{i,t},\;{Pos\;news}_{i,t},\;{Neg\;UGC}_{i,t},{Pos\;UGC}_{i,t}$) is a five-element column vector for firm *i i* at time t*t*, containing the natural logarithm transformation of the DVs; *Φ*_*s*_ are 5×5 matrix of coefficients for *s*-period lagged DVs; *p* is the number of lags. The log transformation means that we can interpret the coefficient estimates as elasticities, a unit of relationship that managers find most actionable and easier to understand. Following prior recall research, we include logarithm of each of the following control variables: complaints, public interest, the volume of UGC, the volume of news [88, 84], sales volume [43, 44, 49, 59], price [14], advertising spending [124], crashes, and product reliability [60]. Leveraging the panel data structure, we further incorporate $f_{i}={\left(f_{1,i},f_{2,i},f_{3,i},f_{4,i},f_{5,i}\right)}^{\prime }$ as unobserved firm-specific fixed effects, characterizing firms’ time-invariant attributes. To control for any industry-wide time trend and seasonality, we include $\xi_{t}=(\xi_{1,t,}\;\xi_{2,t,}\;\xi_{3,t},\xi_{4,t},\xi_{5,t})\prime$ as a column vector of month dummies. Finally, the five-element error vector $\varepsilon_{i,t}=(\varepsilon_{1,i,t},\varepsilon_{2,i,t},\varepsilon_{3,i,t},\varepsilon_{4,i,t},\varepsilon_{5,i,t})\prime (\varepsilon_{1,i,t},\varepsilon_{2,i,t},\varepsilon_{3,i,t}\;)\prime$ satisfies the white noise assumption that $Ε(\varepsilon_{m,i,t})=Ε(\varepsilon_{m,i,t}\varepsilon_{m,i,s})=0$ for any *m* = 1, 2, 3, 4, 5 and *t*≠*s*.

### PVAR estimation procedure

Prior to estimating the PVAR, we completed the following preparatory steps. First, we applied natural log-transformed to variables exhibiting non-normal distributions. Subsequently, the endogeneity issues inherent in dynamic panel data models (i.e., fixed-effects models with lagged DVs as regressors) have been extensively documented in previous studies [143]. To tackle the issues of endogeneity and serial correlation, we implemented the forward orthogonal deviation, also known the Helmert transformation [144]. During this step, the fixed effects are eliminated by concerting all variables in the model into deviations from the forward mean, which involves subtracting the mean of forthcoming observations available for each month. As a result, it maintains homoscedasticity and ensures orthogonality between the forward-differenced variables and lagged DVs [34]. Consequently, we can utilize the lagged regressors as instruments for the forward-differenced variables [40] and employ the coefficients by the system GMM to address the endogeneity issue [34, 144]. Furthermore, employing forward-orthogonal deviations does not induce autocorrelation in the error terms and alleviates us from concerns regarding serial correlation [145]. Following these prerequisites, we proceeded with the standard approaches to estimate our PVAR (see Table 2 for our procedure).

**Table 2:** Overview of PVAR estimation procedure.

Step	Method	Supporting Literature
1. Helmert transformation		[34, 144]
2. Unit root tests	Fisher-type test	[91]
	Im-Pesaran-Shin test	[147]
3. Lag selection	AIC	[34, 153]
	MMSC	[148, 154]
4. Granger causality tests		[42, 149]
5. PVAR main model estimation		[34, 142, 155]
6. PVAR interaction model estimation		[42, 142, 156]
7. Impulse response functions		[34, 42, 142]

## Results

### Unit root tests and lag selection

Because our panel data are unbalanced, we conducted two unit root tests—Fisher-Type [90, 146] and Im-Pesaran-Shin [147]—to verify the absence of unit roots in our five main endogenous variables. The result suggests the absence of a unit root in our panel (see Table A2 in the online S1 Appendix for the results of the unit-root tests). Next, following Dewan and Ramaprasad [40], we calculated AIC for each cross-section and took the modal value of the optimal lag length among all cross-sections. The results indicate that first-order panel VAR (lag 1) is the preferred model. Subsequently, we followed Abrigo and Love [148] to apply the consistent moment and model selection criteria for GMM models to validate further lag 1 selected by AIC (see Table A3 in S1 Appendix for the result of the lag selection).

### Granger causality tests

Given that the PVAR assumes all DV to be endogenous, researchers could conduct Granger causality test to ascertain whether the lagged DVs could predict future values of the same set of DVs within the PVAR system of equations [149]. The outcomes of our Granger causality test (Table 3) reveal that for each corresponding equation, at least one variable exhibits significant Granger causality (*p* < 0.1) (11 of the 20 possible effects using a panel Granger test with one-period lag). This suggests that most variables in our data exert Granger-cause one another. In esstence, the results from the Granger causality tests underscore the necessity of considering the full dynamic system, as in a PVAR, and accounting for the indirect effects within the PVAR system.

**Table 3:** Granger causality tests.

	*Recalls*_*i*,*t*_	*Neg news*_*i*,*t*_	*Pos news*_*i*,*t*_	*Neg UGC*_*i*,*t*_	*Pos UGC*_*i*,*t*_
*Recalls*_*i*,*t*−1_		10.573	3.676	2.550	7.385
		(0.001)	(0.055)	(0.11)	(0.007)
*Neg news*_*i*,*t*−1_	8.587		2.576	4.676	1.170
	(0.003)		(0.109)	(0.031)	(0.279)
*Pos news*_*i*,*t*−1_	35.92	33.623		3.039	0.791
	(0.000)	(0.000)		(0.081)	(0.375)
*Neg UGC*_*i*,*t*−1_	5.607	1.502	12.725		0.613
	(0.018)	(0.220)	(0.000)		(0.434)
*Pos UGC*_*i*,*t*−1_	0.016 (0.899)	0.938	1.007	6.188	
		(0.333)	(0. 316)	(0.013)	

Note: Null hypothesis is that the row variable does not Granger-cause the column variable. Numbers in parentheses are the *p*-values.

### Short-term dynamics of the negativity and the positivity in the news and in UGC, and recalls

Table 4 shows the short-term dynamics among recalls, the negativity in the news, the positivity in the news, the negativity in UGC, and the positivity in UGC. We use this table to test our H_1_ through H_4_. First, we check the results of the regressions with *Recalls* as the DV. Because all the five variables are log-transformed, we can interpret the coefficient estimates as elasticity. The results suggest that the negativity and the positivity in the news and UGC correlate with recalls differently. Specifically, we find that the negativity in the news in a month about a firm’s product defects negatively correlates with its product recalls in the following period, supporting our theoretical argument (H_1_). Stated differently, the result does not support our competing H_1alt_, where we reasoned that the negativity in the news makes managers socially more responsible.

**Table 4:** PVAR estimation results for main associations.

	DV				
Independent Variable	*Recalls*_*i*,*t*_	*Neg news*_*i*,*t*_	*Pos news*_*i*,*t*_	*Neg UGC*_*i*,*t*_	*Pos UGC*_*i*,*t*_
*Recalls*_*i*,*t*−1_	-0.008	0.016**	0.004+	-0.005	-0.008**
	(0.03)	(0.005)	(0.002)	(0.003)	(0.003)
*Neg news*_*i*,*t*−1_	-0.554**	0.023	0.025	-0.050*	-0.022
	(0.188)	(0.034)	(0.016)	(0.023)	(0.021)
*Pos news*_*i*,*t*−1_	2.815***	0.487***	0.064+	-0.081+	-0.040
	(0.470)	(0.084)	(0.033)	(0.046)	(0.045)
*Neg UGC*_*i*,*t*−1_	0.512*	-0.049	-0.065***	0.182***	0.020
	(0.216)	(0.040)	(0.018)	(0.028)	(0.026)
*Pos UGC*_*i*,*t*−1_	-0.031	0.044	-0.021	0.081*	0.151***
	(0.25)	(0.045)	(0.021)	(0.033)	(0.03)
*Complaints*_*i*,*t*−1_	1.712***	0.174*	0.114**	0.021	-0.066
	(0.469)	(0.087)	(0.040)	(0.056)	(0.057)
*Public interest*_*i*,*t*−1_	2.626	0.083	0.352*	0.316	0.702**
	(1.973)	(0.359)	(0.163)	(0.230)	(0.232)
*Volume news*_*i*,*t*−1_	-0.887**	-0.132**	0.077***	0.055+	0.087**
	(0.292)	(0.043)	(0.019)	(0.028)	(0.027)
*Volume UGC*_*i*,*t*−1_	-0.077	-0.039	-0.041**	0.036*	0.041*
	(0.171)	(0.03)	(0.013)	(0.018)	(0.016)
*Sales volume*_*i*,*t*−1_	1.523	0.889***	0.404***	0.242*	-0.287*
	(1.023)	(0.186)	(0.080)	(0.121)	(0.117)
*Price*_*i*,*t*−1_	4.082	3.315***	1.177***	-0.582+	-0.941**
	(3.341)	(0.609)	(0.262)	(0.342)	(0.355)
*Ad spending*_*i*,*t*−1_	-0.764	0.300**	0.100*	0.124*	0.118+
	(0.503)	(0.102)	(0.045)	(0.057)	(0.062)
*Crashes*_*i*,*t*−1_	1.022**	-0.101+	-0.124***	0.020	0.005
	(0.334)	(0.057)	(0.027)	(0.036)	(0.035)
*Reliability*_*i*,*t*−1_	24.351*	16.643***	4.812***	-1.972	-2.647*
	(12.383)	(2.24)	(0.945)	(1.27)	(1.297)
Time dummies	Yes	Yes	Yes	Yes	Yes

**Notes:** The number of observations is 1439, and the number of firms is 22. We use Helmert transformation to remove firm-fixed effects before conducting GMM estimation. Numbers in parentheses are standard errors. Time-fixed effects are included in the estimation, but the coefficient estimates are not shown to conserve space. +*p* < 0.1

*p < 0.05

**p < 0.01

***p < 0.001

The negativity in UGC has an asymmetric association with recalls. Specifically, the negativity in UGC in a month about a firm’s product defects positively correlates with the firm’s number of units recalled in the following month, supporting our H_2alt_. The data thus support our theoretical argument that the public criticism of safety of a firm’s products helps the firm identify defects. We also reason that because regulators and product liability lawyers are known to use UGC in monitoring how proactive/reactive a firm has been in recalling its defective products, the firm has reason to use UGC as a signal of the defect and proactively recall defective units. These two findings contribute to the theory and empirical evidence on how criticism in the two types of earned media—news media and social media—may have an asymmetric association with managerial decisions.

Further, we find that the positivity in news about a firm’s product safety positively correlates with its product recalls in the following month, supporting our H_3_. The finding supports our argument that media praise creates managerial hubris. However, public praise of the safety of a firm’s products does not seem to matter—that is, the data do not support H_4_.

Regarding the control variables, we find that the number of consumer complaints, the number of crashes, and product reliability are positively associated. In contrast, the volume of news about defects in a manufacturer’s vehicles is negatively associated with the number of recalled units.

We also document some interesting patterns in the feedback effect of recalls on the two types of earned media and how they affect each other. For example, in the equation of *Neg news* as the DV, we find that the number of recalled units in a month positively correlates with the negativity in news about the firm’s product defects in the following month. In contrast, in the equation of *Pos UGC* as the DV, the number of units recalled in a month negatively correlates with the positivity in UGC in the following month. These findings suggest a need to consider news *and* UGC (e.g., [47]) to understand interdependencies in phenomena such as product recalls. In addition, in the equation of *Pos news*, the negativity in UGC negatively correlates with the positivity in the news in the following period. These results provide further evidence on the complexity of multichannel communication and imply that an appropriate model is needed to capture this complexity (e.g., [37, 42]).

### Interaction association between news and UGC

We now answer whether news and UGC complement or substitute for each other (i.e., interaction associations) in determining a firm’s recalls. The *Recalls* equation in Table 5 suggests heterogeneous interaction associations between news and UGC. The results show that the negativity in news and the negativity in UGC have a significantly negative interaction association on recalls at the 0.1% level, suggesting a substitution relationship between these two types of earned media. Therefore, our H_5_ is supported. In contrast, the positivity in the news and that in UGC interact positively and significantly (3.648) at the 0.1% level to influence recalls, suggesting a synergistic association between the positivity in the news and that in UGC on the firm’s recalls in the following month. This finding, thus, supports our H_6_. Our results on interaction associations between news and UGC provide further evidence that these two media types’ positive and negative valences play different roles in the recall setting.

**Table 5:** PVAR estimation results for interaction associations.

	Dependent Variable				
Independent Variable	*Recalls*_*i*,*t*_	*Neg news*_*i*,*t*_	*Pos news*_*i*,*t*_	*Neg UGC*_*i*,*t*_	*Pos UGC*_*i*,*t*_
*Recalls*_*i*,*t*−1_	-0.004	0.018***	0.005*	-0.005	-0.008**
	(0.029)	(0.005)	(0.002)	(0.003)	(0.003)
*Neg news*_*i*,*t*−1_	-0.352+	0.051	0.035*	-0.052*	-0.036
	(0.187)	(0.033)	(0.016)	(0.023)	(0.021)
*Pos news*_*i*,*t*−1_	2.465***	0.457***	0.046	-0.063	-0.015
	(0.479)	(0.082)	(0.033)	(0.047)	(0.045)
*Neg UGC*_*i*,*t*−1_	0.647**	-0.036	-0.056**	0.179***	0.020
	(0.221)	(0.04)	(0.018)	(0.028)	(0.026)
*Pos UGC*_*i*,*t*−1_	-0.033	0.046	-0.028	0.070*	0.135***
	(0.272)	(0.044)	(0.021)	(0.032)	(0.029)
*Neg news*_*i*,*t*−1_ *X Neg UGC*_*i*,*t*−1_	-0.713***	-0.150***	-0.069***	0.036	-0.009
	(0.174)	(0.036)	(0.0187)	(0.026)	(0.022)
*Pos news*_*i*,*t*−1_ *X Pos UGC*_*i*,*t*−1_	3.648***	0.592***	0.294***	-0.194**	-0.128*
	(0.527)	(0.090)	(0.040)	(0.062)	(0.055)
Control variables	Yes	Yes	Yes	Yes	Yes
Time dummies	Yes	Yes	Yes	Yes	Yes

**Notes:** The number of observations is 1439, and the number of firms is 22. We use Helmert transformation to remove firm fixed effects before conducting GMM estimation. Numbers in parentheses are standard errors. Control variables and time fixed effects are included in the estimation, but the coefficient estimates are not shown to conserve space. +*p* < 0.1

*p < 0.05

**p < 0.01

***p < 0.001

### Impulse response functions for long-term dynamics

**Fig 1:**
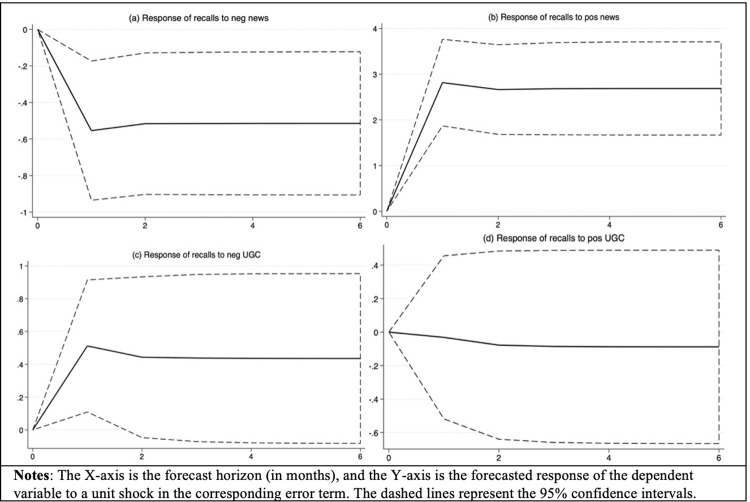
IRF results (negativity and positivity).

Our discussion so far allows us to measure the short-term dynamics. For PVARs, IRFs demonstrate the long-term response of one of the PVAR endogenous variables to the one-unit shock in the value of the other variable [150]. IRFs are defined as the matrices of changes in one variable *i* at a time *t*+*s* for an unexpected one-unit increase in one another variable *j* at time *t* (lagged *s* periods), with all other variables before time *t* held constant. Thus, IRFs allow us to determine whether a shock to one variable will have a permanent or transitory effect on any dependent variable. We conducted IRF analyses with 95% confidence intervals generated from Monte Carlo simulation with 1,000 repetitions. Fig 1 illustrates the results of IRFs for our main–effects model. We are particularly interested in how recalls respond to a shock to the negativity in the news (Fig 1A), the positivity in the news (Fig 1B), the negativity in UGC (Fig 1C), and the positivity in UGC (Fig 1D). The results suggest that both the negativity in news and the positivity in news have long-term associations with recalls (Fig 1A and 1B), and the pattern of these long-term associations is quite different. In contrast, we do not find evidence supporting long-term associations of either the negativity in UGC (Fig 1C) or the positivity in UGC (Fig 1D) on recalls.

For example, Fig 1A illustrates that the change in recalls in response to a shock in the negative news is negative. This association peaks around month 1 and significantly differs from zero from month 1 to month 6. On the other hand, the change in recalls in response to a shock in the positivity in the news (Fig 1B) reaches a peak around month 1 and remains positive over time. We observe similarly asymmetric patterns in the associations of the negativity in UGC and the positivity in UGC. For example, the negativity in UGC positively correlates with recalls in month 1 (Fig 1C). However, this association is not significantly different from zero from month 2 to month 6, suggesting a transitory association of the negativity in UGC on recalls. This finding is also consistent with characterizing user-generated tweets as a fast-paced way to discover new content and see what trending is [42]. Finally, we find no evidence supporting short- and long-term associations of the positivity in UGC on recalls. Overall, IRFs show a graphical representation of how the system evolves and can provide important implications for managers who monitor the earned media. For example, because the negativity and the positivity in the news take around one month to reach their peak predictive association on recalls, managers should visit their news response strategies every month to better manage their news media reputation.

### Additional analyses

Our results provide insights into the different associations of news and UGC play in a managerial recall decision. These associations may vary by recall characteristics. Specifically, we contend that the proposed relationships will be contingent upon the level of recall severity, such that these relationships will show consistent patterns, such as the main results for high-severity recalls but demonstrate the different patterns for low-severity recall. Prior research suggests that high-severity recalls involve less managerial discretion as consumers’ lives and the firm’s liability risk are at stake, and the need for a recall is more apparent [49]. Therefore, high-severity recalls represent more objective product quality issues. Thus, we expect to see the relationships between news, UGC, and recalls for high-severity recalls, which characterize low managerial discretion. On the other hand, product defects with less immediate safety concerns are usually less evident in the public domain. The recall decision will become more challenging because managers need to involve high managerial discretion with more subjective judgment [49]. Thus, we expect to see different patterns for low-severity recalls. Following Astvansh and Eshghi [4] and Liu and Shankar [151], we classify a recall as a high-severity recall if keywords such as *injury*, *crash*, *death*, or *fire* appear in the consequence summary of the NHTSA recall data. A recall characterized by these conditions involves an immediate safety concern [151]. Otherwise, we treat the recall as low severity. Based on this classification, we differentiate two recall variables: high-severity recalls and low-severity recalls.

Tables 6 and 7 show the short-term dynamics for high-severity recalls and low-severity recalls, respectively. The results of high-severity recalls (Table 6) are consistent with the main results (Table 4). For example, the negativity in the news negatively correlates with the number of units recalled as part of high-severity recalls. In contrast, the positivity in news and the negativity in UGC positively correlate with the number of units recalled as part of high-severity recalls. In contrast, we observe very different patterns for low-severity recalls (see Table 7). For example, the positivity in the news is positively correlated with low-severity recalls, which has the opposite direction as the corresponding one for high-severity recalls.

**Table 6:** PVAR estimation results for high-severity recalls.

	Dependent Variable				
Independent Variable	*High severity recalls*_*i*,*t*_	*Neg news*_*i*,*t*_	*Pos news*_*i*,*t*_	*Neg UGC*_*i*,*t*_	*Pos UGC*_*i*,*t*_
*High severity recalls*_*i*,*t*−1_	-0.029	0.015**	0.004+	-0.004	-0.007**
	(0.029)	(0.005)	(0.002)	(0.003)	(0.003)
*Neg news*_*i*,*t*−1_	-0.594**	0.023	0.022	-0.032	-0.023
	(0.188)	(0.034)	(0.015)	(0.023)	(0.021)
*Pos news*_*i*,*t*−1_	1.728***	0.411***	0.056+	-0.067	0.003
	(0.455)	(0.084)	(0.033)	(0.047)	(0.045)
*Neg UGC*_*i*,*t*−1_	0.548*	-0.045	-0.060***	0.173***	0.032
	(0.214)	(0.041)	(0.018)	(0.028)	(0.026)
*Pos UGC*_*i*,*t*−1_	0.089	0.068	-0.001	0.085**	0.154***
	(0.243)	(0.046)	(0.021)	(0.033)	(0.03)
Control variables	Yes	Yes	Yes	Yes	Yes
Time dummies	Yes	Yes	Yes	Yes	Yes

**Notes:** The number of observations is 1439, and the number of firms is 22. We use Helmert transformation to remove firm-fixed effects before conducting GMM estimation. Numbers in parentheses are standard errors. Control variables and time-fixed effects are included in the estimation, but the coefficient estimates are not shown to conserve space. +*p* < 0.1

*p < 0.05

**p < 0.01

***p < 0.001

**Table 7:** PVAR estimation results for low-severity recalls.

	Dependent Variable				
Independent Variable	*Low severity recalls*_*i*,*t*_	*Neg news*_*i*,*t*_	*Pos news*_*i*,*t*_	*Neg UGC*_*i*,*t*_	*Pos UGC*_*i*,*t*_
*Low severity recalls*_*i*,*t*−1_	0.057*	0.016**	0.004+	-0.005	-0.008**
	(0.029)	(0.005)	(0.002)	(0.003)	(0.003)
*Neg news*_*i*,*t*−1_	0.004	0.023	0.025	-0.050*	-0.022
	(0.022)	(0.034)	(0.016)	(0.023)	(0.021)
*Pos news*_*i*,*t*−1_	-0.084*	0.487***	0.064+	-0.081+	-0.040
	(0.024)	(0.084)	(0.033)	(0.046)	(0.045)
*Neg UGC*_*i*,*t*−1_	-0.008	-0.049	-0.065***	0.1852***	0.020
	(0.024)	(0.040)	(0.018)	(0.028)	(0.026)
*Pos UGC*_*i*,*t*−1_	-0.009	0.044	-0.021	0.081*	0.151***
	(0.028)	(0.045)	(0.021)	(0.033)	(0.03)
Control variables	Yes	Yes	Yes	Yes	Yes
Time dummies	Yes	Yes	Yes	Yes	Yes

**Notes:** The number of observations is 1439, and the number of firms is 22. We use Helmert transformation to remove firm-fixed effects before conducting GMM estimation. Numbers in parentheses are standard errors. Control variables and time-fixed effects are included in the estimation, but the coefficient estimates are not shown to conserve space. +*p* < 0.1

*p < 0.05

**p < 0.01

***p < 0.001

Finally, we conduct interaction and IRF analyses for high and low-severity recalls. The result for high-severity recalls shows consistent patterns like the main results, while that for low-severity recalls demonstrates different patterns. Tables A4 and A5 in the online S1 Appendix present these results, and Figures A1 and A2 in S1 Appendix depict the IRF results for high- and low-severity recalls, respectively.

## Discussion

This study examines the dynamic interdependencies of the negativity and the positivity in news and user-generated content on a firm’s product recalls. We believe our findings have important theoretical implications for researchers and practical implications for managers.

### Theoretical implications

The multidisciplinary literature on product quality failures and recalls has documented various factors determining a firm’s recalls in any period. However, in general, these factors belong to the firm’s *internal* environment, including product characteristics, supply chain, corporate governance, and the industry. We aim to extend this literature by asking: Does a firm’s earned media influence its recalls? In answering this question, we highlight the relationship between a firm’s product-market decisions and the behavior of its stakeholders outside the firm’s boundaries, in this case, journalists reporting news and users creating and disseminating content on social media platforms.

Research at the intersection of earned media and business has examined how these different types of earned media may affect firm outcomes. However, most of this research has evolved in silos [51], with management researchers focusing on news organizations and information systems and marketing researchers paying more attention to UGC. By including both sources of earned media in this study and modeling their dynamic relationships, we offer a more holistic and nuanced picture of how earned media affect firms. We show that news and UGC have asymmetric associations and that their associations persist for different durations. In addition, the valence of earned media determines whether it complements or substitutes for the other, leading us to question the popular notion that UGC can replace news [39]. Lastly, in assessing the impacts of earned media on an outcome that is consequential to both journalists and civil society, we extend the accumulated knowledge that shows that the two types of earned media affect sales and other marketing outcomes, albeit separately [40, 51].

Our findings about the feedback loops [37] between news and UGC further media research as well. Specifically, the finding that negative UGC negatively correlates with positive news supports Stephen and Galak’s [51: 626] assertion that “traditional media [drives] social media. However, the reverse is also plausible and, arguably, more likely in certain settings.” Lastly, we find that the relationship among news, UGC, and recalls is not universal but is contingent upon the severity degree of recalls, which involve different degrees of managerial discretion. Given the heterogeneous nature of recalls, using managerial discretion as a lens contributes to the emerging evidence on the role of managerial discretion (e.g., [49]) by better understanding the boundary of how two stakeholders would influence recalls.

### Managerial implications

We believe that managers can benefit from our finding of asymmetric interdependencies. That is, the negativity in the news decreases firm recalls, while the negativity in UGC increases firm recalls. Interestingly, the elasticities of negative news (–0.554%) and negative UGC (0.512%) are comparable in magnitude but opposite in direction. Further, while the positivity in news positively correlates with a firm’s recalls, the positivity in UGC does not have a significant impact; the latter finding being contrary to our hypothesis. These findings suggest that managers should be watchful of negative earned media and avoid simply basking in the glory of positive media. The need for vigilance seems even more consequential given our finding that the negativity in the news and the negativity in UGC substitute for each other. In contrast, the positivity in the news and the positivity in UGC complement each other.

In addition, we document the longer-term associations of the negativity and the positivity in the news with a firm’s recalls. We find that the association of the negativity in news peaks in the first month and stays negative over time. On the other hand, the association of the positivity in news peaks in the first month and stays positive over time. Therefore, managers should stay vigilant about negative and positive news each month. Further, the positive association of the negativity in UGC also peaks in the first month but becomes negligible in the second month. The finding is consistent with the popular notion that UGC peaks and fades within a few days. Managers should not discount the association of negative UGC in the first few days but rather try to devise strategies to leverage its benefits and lower its costs.

Lastly, we document interesting feedback associations of earned media. For example, the negativity in UGC negatively correlates with positivity in news in the following period. In contrast, the positivity in news negatively correlates with the negativity in UGC in the following period. These results imply that managers should have a comprehensive plan to monitor and respond to different types of earned media.

### Limitations and future research

We note several limitations of our study, each of which merits future research. First, a common theme in business communications research is the interdependency among earned media (e.g., news, customers’ reviews of a firm’s offerings), paid media (i.e., advertising on social media platforms, Internet search keywords, Internet displays, and email), and owned media (e.g., press releases, firm-generated content on social media platforms, executives’ blogs on a firm’s website) [37, 51]. Our focus in this article has been on the interrelationships between the content of the two types of earned content—news and UGC—while controlling for advertising expenditure. However, like other researchers (e.g., [51]), we acknowledge the blurred line between earned and paid content. Future research can overcome this blurring by classifying content as social versus traditional/mass. Future research could, for example, separate news into two variables: news disseminated by news organizations on social media platforms versus that distributed through mass media. Similarly, content on social media platforms could be earned (UGC) versus owned (FGC) [152].

Second, to keep our research focus, we did not explore how the actions of the focal firm and its rivals can moderate the associations of news and UGC on recalls [35, 129]. Future research could examine, for example, whether a firm’s apology advertising and responses to tweets moderate the main associations of news and UGC.

Third, because we exclusively studied automobile failures and used data from Twitter only, researchers may want to extend the scope of this inquiry. Future studies may test the generalizability of our findings by studying failures in services and other product categories (e.g., medical devices), and sourcing UGC from Facebook, Instagram, and review websites and apps. Fifth, we incorporated organizational perception (media reputation and public reputation) as a theoretical mechanism. Further research could collect data on these mechanism variables and estimate mediation models. More broadly, researchers could develop a theory and test when, how, and why earned media affects recalls.

Moreover, although we have followed several prior recall research (e.g., [44, 49, 124] to control several firm- and recall-related characteristics in our models, our control sets may exclude some other possible control variables due to data limitations. Further research could consider these possible control variables when the data becomes available. Finally, our data covers the period from 2009 to 2015. Twitter (now X) has changed its data collection policy significantly since then. Due to this limitation, we cannot collect more recent Twitter data. Further studies may consider collecting more recent data to examine if there are any interesting patterns in the dynamic interdependencies once X relaxes its data collection policy.

## Supporting information

S1 File (DTA)

S1 Appendix (DOCX)
