# Supplementary material for: The dynamic interdependencies among the negativity and the positivity in news and user-generated content about safety in a firm’s products and the firm’s product recalls
Source: PLoS One. 2024 Aug 8;19(8):e0305287. doi: 10.1371/journal.pone.0305287 (PMC11309489; doi:10.1371/journal.pone.0305287)
Supplement: S1 Appendix — (DOCX) [file pone.0305287.s002.docx]

**The Dynamic Interdependencies Among the Negativity and the Positivity in News and User-Generated Content about Safety in a Firm’s Products and the Firm’s Product Recalls**

# **Online Appendix**

## **Table A1: Correlation Coefficients**

| Variables | (1) | (2) | (3) | (4) | (5) | (6) | (7) | (8) | (9) | (10) | (11) | (12) | (13) | (14) |
| --- | --- | --- | --- | --- | --- | --- | --- | --- | --- | --- | --- | --- | --- | --- |
| (1) Recalls | 1.000 |  |  |  |  |  |  |  |  |  |  |  |  |  |
| (2) Neg news | 0.166 | 1.000 |  |  |  |  |  |  |  |  |  |  |  |  |
| (3) Pos news | 0.120 | 0.636 | 1.000 |  |  |  |  |  |  |  |  |  |  |  |
| (4) Neg UGC | -0.066 | -0.093 | -0.079 | 1.000 |  |  |  |  |  |  |  |  |  |  |
| (5) Pos UGC | -0.071 | -0.153 | -0.107 | 0.269 | 1.000 |  |  |  |  |  |  |  |  |  |
| (6) Complaints | 0.263 | 0.201 | 0.211 | -0.021 | -0.056 | 1.000 |  |  |  |  |  |  |  |  |
| (7) Google_trends | 0.066 | 0.158 | 0.098 | 0.093 | -0.045 | 0.171 | 1.000 |  |  |  |  |  |  |  |
| (8) Volume of news | 0.275 | 0.230 | 0.199 | -0.074 | -0.072 | 0.409 | 0.123 | 1.000 |  |  |  |  |  |  |
| (9) Volume of UGC | 0.180 | 0.159 | 0.123 | -0.117 | -0.132 | 0.305 | -0.030 | 0.295 | 1.000 |  |  |  |  |  |
| (10) Sales volume | 0.212 | 0.208 | 0.183 | -0.021 | -0.084 | 0.742 | 0.215 | 0.095 | 0.238 | 1.000 |  |  |  |  |
| (11) Price | -0.041 | 0.146 | 0.088 | 0.026 | -0.045 | -0.272 | 0.352 | 0.043 | -0.106 | -0.326 | 1.000 |  |  |  |
| (12) Ad spending | 0.133 | 0.200 | 0.162 | -0.048 | -0.088 | 0.607 | 0.172 | 0.103 | 0.212 | 0.853 | -0.289 | 1.000 |  |  |
| (13) Crash | 0.204 | 0.158 | 0.150 | -0.029 | -0.041 | 0.774 | 0.137 | 0.583 | 0.402 | 0.454 | -0.141 | 0.408 | 1.000 |  |
| (14) Reliability | -0.019 | 0.074 | 0.001 | 0.035 | -0.040 | -0.133 | 0.214 | 0.037 | -0.034 | -0.092 | 0.324 | -0.054 | -0.014 | 1.000 |
|  | | | | | | | | | | | | | | |

**1. Unit Root Tests**

We conducted two different types of unit root tests to verify the absence of unit roots in our panel data: Fisher-Type (Choi [2001](https://econpapers.repec.org/article/eeejimfin/v_3a20_3ay_3a2001_3ai_3a2_3ap_3a249-272.htm)) and Im-Pesaran-Shin (Im 2003) tests. The results for both tests are shown in Table OS 1. All p-values are smaller than 0.01, suggesting that there is no unit root in our panel. Full variable correlation is also shown in Table OS 2.

## **Table A2: Fisher-type and Im-Pesaran-Shin Unit-Root Tests**

| **Variable** | **Inverse chi-squared obs.** | **Inverse normal** | **Inverse logit t** | **Modified inverse chi-squared** | **W-t-bar** |
| --- | --- | --- | --- | --- | --- |
| Recalls | 290.11 (0.000) | -12.72 (0.000) | -16.98 (0.000) | 26.24 (0.000) | -24.65 (0.000) |
| Neg news | 288.71 (0.000) | -12.27 (0.000) | -14.05 (0.000) | 26.08 (0.000) | -27.15 (0.000) |
| Pos news | 332.81 (0.000) | -14.64 (0.000) | -19.6 (0.000) | 30.78 (0.000) | -26.67 (0.000) |
| Neg UGC | 410.54 (0.000) | -13.4 (0.000) | -16.97 (0.000) | 39.07 (0.000) | -29.89 (0.000) |
| Pos UGC | 398.47 (0.000) | -16.92 (0.000) | -23.51 (0.000) | 37.78 (0.000) | -33.16 (0.000) |

**Notes:** The second to the fifth column are results of the Fisher-type test; the sixth column represents results of the Im-Pesaran-Shin test.

Numbers in parentheses are *p*-value. The null hypothesis – that all panels contain unit roots – is rejected for all variables.

**2. Lag Selection**

We leveraged moment and model selection criteria (MMSC) to double check the validity of lag 1 that was selected by AIC. Andrews and Lu ([2001](https://www.sciencedirect.com/science/article/abs/pii/S0304407600000774)) proposed consistent MMSC for GMM models based on Hansen’s ([1982)](https://www.jstor.org/stable/1912775?seq=1#metadata_info_tab_contents) statistic of over-identifying restrictions. Their proposed MMSC are analogous to various commonly used maximum likelihood-based model selection such as AIC. Table OS 3 report the results of MMSC for our main PVAR model. The criterion is to select the test with the smallest MMSC-Bayesian information criterion (MBIC), MMSC-Akaike’s information criterion (MAIC), and MMSC-Hannan and Quinn information criterion (MQIC). The results indicate that the length of lag 1 is the preferred number for our PVAR model.

## **Table A3: Lag Selection of the Main PVAR**

| **Lag** | **CD** | **J** | **J p-value** | **MBIC** | **MAIC** | **MQIC** |
| --- | --- | --- | --- | --- | --- | --- |
| 1 | 0.321 | 562.3 | 0.126 | -3052.10 | -487.75 | -1463.48 |
| 2 | 0.469 | 524.67 | 0.215 | -2917.55 | -475.30 | -1404.58 |
| 3 | 0.523 | 487.95 | 0.331 | -2782.16 | -462.03 | -1344.84 |
| 4 | 0.291 | 497.87 | 0.058 | -2600.13 | -402.10 | -1238.45 |

**3. Interaction and IRF Analyses for High and Low Severity Recalls**

Tables OS.4 and OS.5 show results of interaction analyses for high and low severity recalls, respectively. These results suggest again that results for high severity recalls (see Table OS 4) have consistent patterns like the main interaction results (see Table 5 in the main paper). For example, we also find a substitution relationship between the negativity in the news and UGC and a synergistic effect between the positivity in news and that in UGC on the firm’s high severity recalls while there is only a synergistic effect between the positivity in news and that in UGC on the firm’s low severity recalls (see Table OS 5).

## **Table A4: PVAR Estimation Results for Interaction Effects (High Severity Recalls)**

|  | **Dependent Variable** | | | | |
| --- | --- | --- | --- | --- | --- |
| **Independent Variable** | ${High severity recalls}_{i,t-1}$ | ${Neg news}_{i,t}$ | ${Pos news}_{i,t}$ | ${Neg UGC}_{i,t}$ | ${Pos UGC}_{i,t}$ |
| ${High severity recalls}_{i,t-1}$ | -0.004  (0.029) | 0.016***  (0.005) | 0.004*  (0.002) | -0.004  (0.003) | -0.008**  (0.003) |
| ${Neg news}_{i,t-1}$ | -0.440*  (0.188) | 0.023  (0.034) | 0.016  (0.016) | -0.040+ (0.024) | -0.035+  (0.021) |
| ${Pos news}_{i,t-1}$ | 1.353**  (0.477) | 0.395***  (0.083) | 0.035  (0.033) | -0.077  (0.048) | -0.016  (0.045) |
| ${Neg UGC}_{i,t-1}$ | 0.781***  (0.226) | -0.020  (0.041) | -0.044*  (0.018) | 0.172***  (0.029) | 0.044+  (0.027) |
| ${Pos UGC}_{i,t-1}$ | 0.366  (0.267) | 0.075+  (0.044) | 0.012  (0.020) | 0.124***  (0.033) | 0.199***  (0.030) |
| ${Neg news}_{i,t-1}X{Neg UGC}_{i,t-1}$ | -0.557**  (0.177) | -0.139***  (0.036) | -0.069***  (0.017) | 0.035  (0.026) | -0.018  (0.022) |
| ${Pos news}_{i,t-1} X{Pos UGC}_{i,t-1}$ | 3.880***  (0.527) | 0.635***  (0.093) | 0.323***  (0.039) | -0.228***  (0.063) | -0.058  (0.056) |
| Control variables | Yes | Yes | Yes | Yes | Yes |
| Time dummies | Yes | Yes | Yes | Yes | Yes |
| **Notes:** The number of observations is 1439, and the number of firms is 22. We use Helmert transformation to remove firm fixed effects before conducting GMM estimation. Numbers in parentheses are standard errors. Control variables and time fixed effects are included in the estimation, but the coefficient estimates are not shown to conserve space. +*p* < 0.1; **p* < 0.05; ***p* < 0.01; ****p* < 0.001 | | | | | |

## **Table A5: PVAR Estimation Results for Interaction Effects (Low Severity Recalls)**

|  | **Dependent Variable** | | | | |
| --- | --- | --- | --- | --- | --- |
| **Independent Variable** | ${Low severity recalls}_{i,t-1}$ | ${Neg news}_{i,t}$ | ${Pos news}_{i,t}$ | ${Neg UGC}_{i,t}$ | ${Pos UGC}_{i,t}$ |
| ${Low severity recalls}_{i,t-1}$ | 0.054+  (0.029) | -0.001  (0.013) | 0.000  (0.005) | -0.014  (0.009) | -0.003  (0.006) |
| ${Neg news}_{i,t-1}$ | 0.004  (0.023) | 0.021  (0.032) | -0.016  (0.015) | -0.007  (0.022) | -0.012  (0.020) |
| ${Pos news}_{i,t-1}$ | -0.108*  (0.042) | 0.279***  (0.077) | 0.201***  (0.032) | 0.033  (0.046) | 0.003  (0.041) |
| ${Neg UGC}_{i,t-1}$ | -0.002  (0.023) | -0.048  (0.037) | -0.021  (0.016) | 0.161***  (0.028) | 0.044+  (0.025) |
| ${Pos UGC}_{i,t-1}$ | 0.014  (0.027) | 0.073+  (0.043) | 0.006  (0.019) | 0.062*  (0.032) | 0.164***  (0.028) |
| ${Neg news}_{i,t-1}X{Neg UGC}_{i,t-1}$ | -0.019  (0.024) | -0.052  (0.033) | -0.018  (0.015) | 0.011  (0.027) | -0.013  (0.022) |
| ${Pos news}_{i,t-1} X{Pos UGC}_{i,t-1}$ | 0.177**  (0.054) | 0.503***  (0.087) | 0.211***  (0.039) | -0.146*  (0.060) | 0.016  (0.053) |
| Control variables | Yes | Yes | Yes | Yes | Yes |
| Time dummies | Yes | Yes | Yes | Yes | Yes |
| **Notes:** The number of observations is 1439, and the number of firms is 22. We use Helmert transformation to remove firm fixed effects before conducting GMM estimation. Numbers in parentheses are standard errors. Control variables and time fixed effects are included in the estimation, but the coefficient estimates are not shown to conserve space. +*p* < 0.1; **p* < 0.05; ***p* < 0.01; ****p* < 0.001 | | | | | |

We finally conduct IRFs analyses for high and low severity recalls (see Figures OS 1 and 2 for high severity recalls and low severity recalls, respectively). When we compare these results with the main IRFs results (Figure 1 in the main paper), we also observe very interesting patterns. We find that IRFs results for high severity recalls show the consistent patterns like the main IRFs results. For example, like the main IRFs results, the negativity in news has a long-term negative effect on high severity recalls while the positivity in news has a long-term positive effect on high severity recalls. Interestingly, we document that the negativity in UGC has a long-term positive effect on high severity recalls while we only find that the negativity in UGC a short-term positive effect on recalls in our main IRFs analysis. On the other hand, we have a very different pattern for low severity recalls, where we only document that the positivity in news has a long-term negative effect on low severity recalls. To conclude, our analyses on high and low severity recalls suggest that the relationships between news, UGC, and recalls are contingent upon the level of recall severity which involves different degree of managerial discretion in making the recall decision.

## **Figure A1: IRF Results for High Severity Recalls**


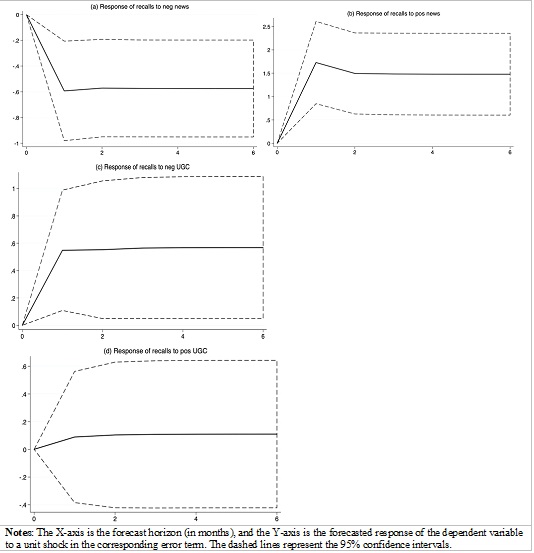


## **Figure A2: IRF Results for Low Severity Recalls**


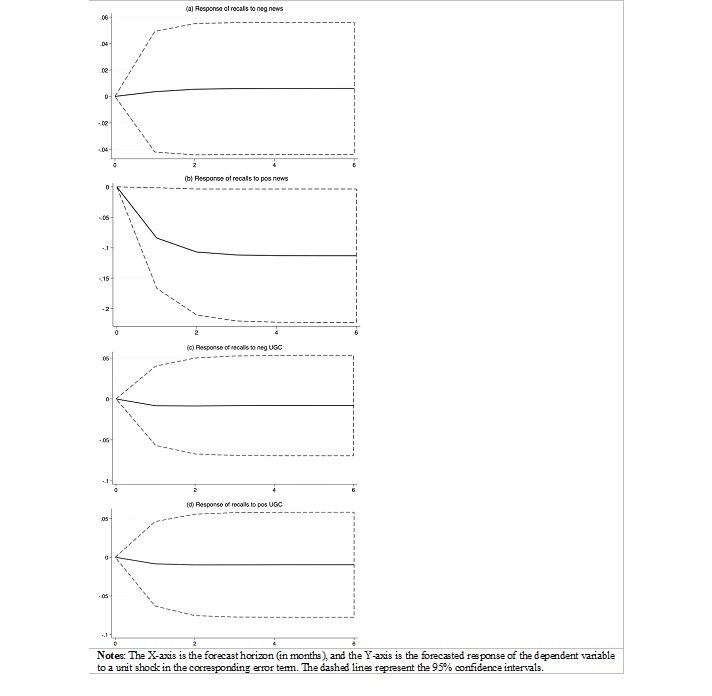


# **References for Online Appendix**

Andrews, D. W., & Lu, B. (2001). Consistent model and moment selection procedures for gmm estimation with application to dynamic panel data models. *Journal of Econometrics*. 101(1):123–164.

Choi, I. (2001). Unit root tests for panel data. *Journal of International Money and Finance*. 20(2):249–272.

Hansen, L. P. (1982). Large sample properties of generalized method of moments estimators. *Econometrica: Journal of the Econometric Society*. 50(4):1029–1054.

Im, K. S., Pesaran, M. H., & Shin, Y. (2003). Testing for unit roots in heterogeneous panels. *Journal of Econometrics*. 115(1):53–74.
